# Infants and elderlies are susceptible to zinc deficiency

**DOI:** 10.1038/srep21850

**Published:** 2016-02-25

**Authors:** Hiroshi Yasuda, Toyoharu Tsutsui

**Affiliations:** 1 La Belle Vie Research Laboratory, Tokyo 103-0006, Japan

## Abstract

The importance of zinc for human health has been recognized since the early 1960s, but today there is little concern about zinc deficiency in developed countries. In this study, we measured the zinc concentration in hair from 28,424 Japanese subjects (18,812 females and 9,612 males) and found that 1,754 subjects (6.17%) had zinc concentrations lower than 2 standard deviations (86.3 ppm) below the control reference range, which qualifies as zinc deficiency. In particular, a considerable proportion of elderlies and children (20% or more) were found to have marginal to severe zinc deficiency. A zinc concentration of 9.7 ppm was the lowest observed in a 51-year-old woman; this concentration was approximately 1/13 of the mean reference level. The prevalence of zinc deficiency in adults increased with aging to a maximum of 19.7% by the 8^th^ decade of life, and decreased to 3.4% above 90-year-old. The proportion of zinc deficiency in infants 0–4 years was 36.5% in males and 47.3% in females; these percentages were higher than the maximum prevalence in elderly subjects. These findings suggest that infants and elderlies are prone to zinc deficiency and that intervention of zinc deficiency is necessary for normal human development, health and longevity.

The importance of zinc for human health has been recognized since the early 1960s^1^,^2^. Zinc is an essential micronutrient required for the activity of more than 300 enzymes and 1,000 transcription factors and for the control of genetic expression. It also plays important roles in nucleic acid/protein synthesis, cell replication, and tissue growth and repair^1^,^2^,^3^,^4^. Zinc deficiency is known to be associated with various pathological conditions, including impaired immunity, delayed wound healing, retarded growth, neural development disorders and degenerative diseases^1^,^4^.

The use of serum samples alone to assess zinc nutritional status has limitations because serum zinc levels are influenced by factors other than dietary zinc intake, including hypoalbuminemia, infection, acute stress, pregnancy and the use of oral contraceptive agents, all of which can lower serum zinc levels. In addition, serum zinc levels are subject to diurnal variation and are influenced by the fasting status of subjects^5^. Serum zinc levels can be normal even in states of mild deficiency because zinc works primarily as an intracellular nutrient^6^,^7^.

Recent advances in highly sensitive and reliable trace element analysis methods using inductively coupled plasma mass spectrometry (ICP-MS) have enabled the detection of chronic essential mineral deficiencies in the human body. These analyses have shown that human whole blood mineral concentrations are reflected in hair and nail specimens^8^,^9^. Thus, clinical metallomics studies with reliable ICP-MS methods have been used to investigate the association of several diseases and symptoms with the dynamics of trace bio-elements including toxic metals and essential minerals^10^,^11^,^12^,^13^,^14^,^15^,^16^,^17^,^18^,^19^,^20^.

In this cohort study investigating zinc deficiency, we examined human scalp hair zinc concentrations in 28,424 Japanese subjects with ages ranging from 0 to 100 years.

## Results

**Figure 1:**
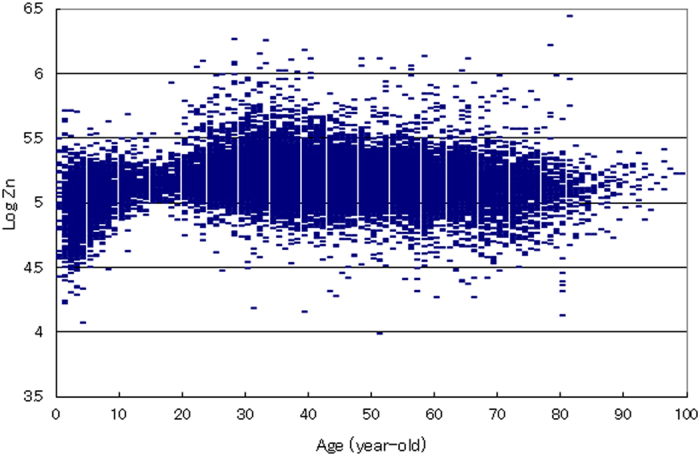
Relationship between hair zinc concentration and age in 28,424 Japanese subjects. The association between the log of hair zinc concentration and age in Japanese subjects aged 0–100 years (N = 28,424) is shown. Each point represents the corresponding age and log of zinc concentration for the respective subject. The ordinate indicates the log of hair zinc concentration (ng/g hair: ppb).

**Figure 2:**
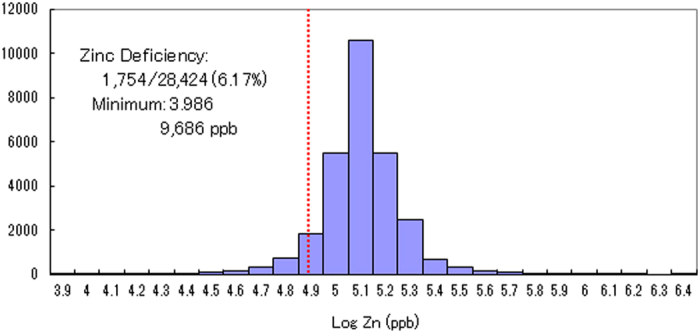
Histogram of log zinc concentration in Japanese subjects (N = 28,424). Hair zinc concentrations for 28,424 subjects are shown in the log plot. The numbers on the abscissa indicate the log of hair zinc concentrations (ng/g hair: ppb). The height of each rectangle represents the frequency in the respective class interval of the log of hair zinc concentration. The dotted vertical line represents the −2 S.D. (standard deviation) level of the control reference range for hair zinc concentrations.

We measured hair zinc concentrations in 28,424 Japanese subjects (18,812 females and 9,612 males) aged 0–100 years (Fig. 1). The log of the zinc concentration was normally distributed with tailing in the lower range. A total of 1,754 of the 28,424 subjects (6.17%) were found to have zinc concentrations lower than 2 standard deviations (S.D.) below the control reference range (86.3–193 μg/g hair (ppm); geometric mean = 129 ppm), which is considered zinc deficiency (Fig. 2). The lowest zinc concentration measured was 9.69 ppm and was detected in a 51-year-old woman; this concentration corresponded to approximately 1/13 of the mean reference level.

**Figure 3:**
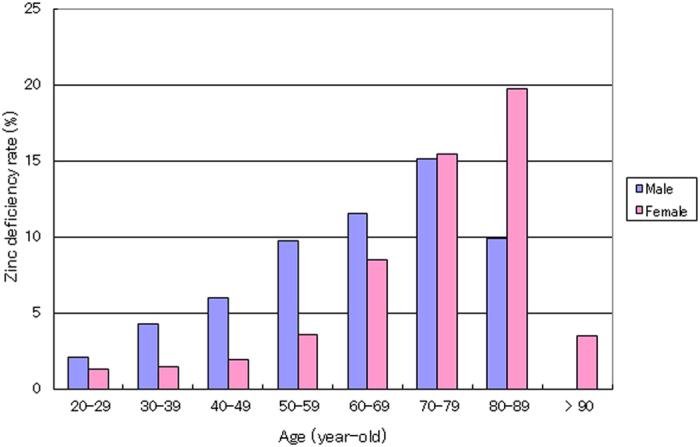
Age-related increase in prevalence of zinc deficiency in adult subjects. The association of zinc deficiency rate with age group (20–100 years old) in adult subjects (N = 25,500) is shown. The height of each rectangle represents the rate of zinc deficiency in the respective age group. The ordinate indicates the zinc deficiency rate (%).

**Figure 4:**
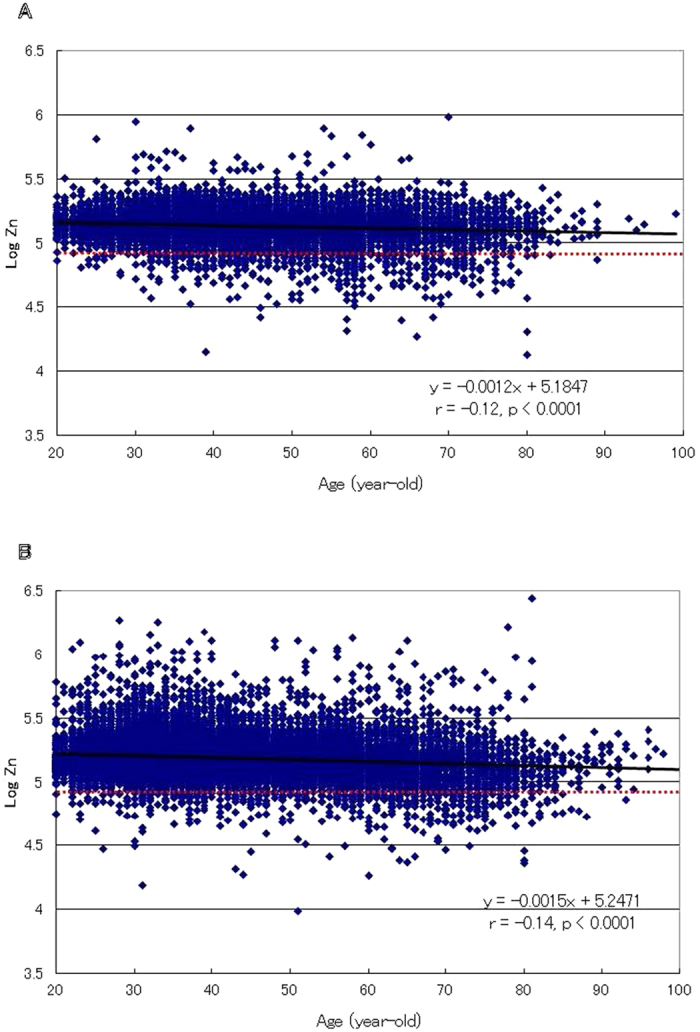
Relationship between zinc concentration and age in male (**A**) and female (**B**) adults. The association of the log of hair zinc concentration with age in male (N = 7,509) and female (N = 17,991) adults aged 20–100 years is shown. Each point represents the corresponding age and log of zinc concentration of the respective subject. The ordinate indicates the log of hair zinc concentration (ng/g hair: ppb). The dotted horizontal line represents the −2 S.D. (standard deviation) level of the control reference range for hair zinc concentrations. A significant inverse relationship between hair zinc concentration and age in the male (**A**) and female (**B**) adults is shown (r = −0.12 and −0.14, respectively, p < 0.0001).

The prevalence of zinc deficiency in the adult male group increased with increasing age, and when assessed by decade it ranged from 2.0% at the 2^nd^ decade to 4.2, 6.0, 9.7, 11.6% and reached 15.1% by the 7^th^ decade; the rate then decreased to 9.3% or less for ages over 80 years (Fig. 3). The zinc deficiency rate in the adult female group also increased by decade from 1.3% at the 2^nd^ decade to 1.5, 1.9, 3.6, 8.5, 15.4% and reached a maximum of 19.7% by the 8^th^ decade; it then decreased to 3.4% for ages above 90 years (Fig. 3). A significant (p < 0.001) inverse correlation between the log of the zinc concentration and age (r = −0.12 and −0.14 for male and female groups, respectively) was observed (Fig. 4A,B).

**Figure 5:**
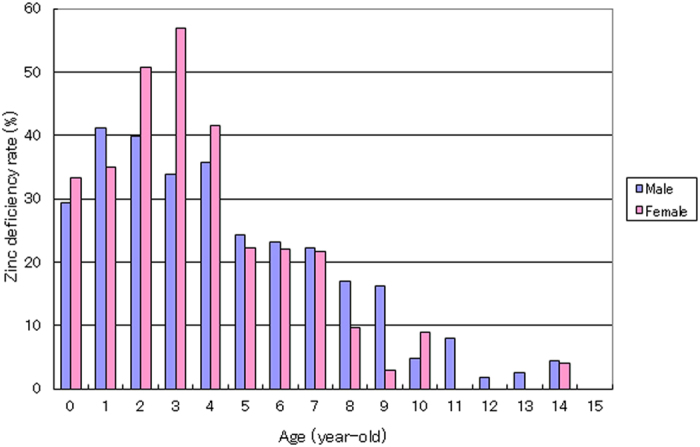
Age-related zinc deficiency rate in children aged 0–15 years. The incidence of zinc deficiency in every age group is shown for 2,685 children (1,998 males and 687 females) aged 0–15 years. The height of each rectangle represents the rate of zinc deficiency in the respective age group.

**Figure 6:**
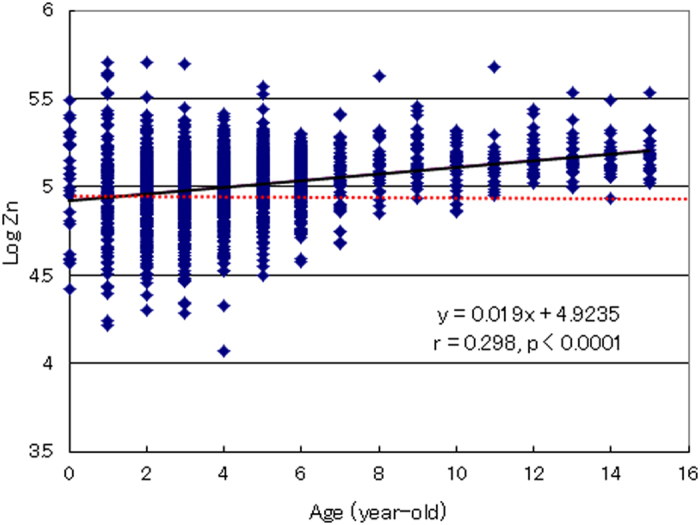
Relationship between zinc concentration and age in children. The association of the log of hair zinc concentration with age in children aged 0–15 years (N = 2,685) is shown. Each point represents the corresponding age and log of zinc concentration in the respective subject. The dotted horizontal line represents the −2 S.D. (standard deviation) level of the control reference range for hair zinc concentrations. A significant correlation between the log of zinc concentrations and age was observed in the children (r = 0.298, p < 0.0001).

The prevalence of zinc deficiency in the male and female groups of children aged 0–9 years was 29.9% and 33.8%, respectively; these rates were both higher than the maximum of 19.7% observed in the adult groups. In particular, in infants aged 0–4 years, the prevalence of zinc deficiency was 36.5 and 47.3% in males and females, respectively, and higher rates, over 50%, were observed in the age group including 2- and 3-year-old (Fig. 5). In addition, a highly significant correlation between the zinc concentration and age was observed in the child group (r = 0.298, p < 0.0001), with a plateau at ages over 10 years (Fig. 6). These findings indicate that infants are more prone to zinc deficiency than elderly individuals, which suggests that an early intervention to correct zinc deficiency is necessary for normal child development and health.

## Discussion

The importance of zinc in human nutrition and health has been recognized since the early 1960s^1^,^2^. Because zinc is required for the synthesis and repair of DNA, RNA and proteins, this micronutrient appears to affect the basic biochemical and physiological processes involved in cell growth, cell division, cell differentiation, development and aging^1^,^2^,^3^,^4^. Clinical signs of zinc deficiency include acrodermatitis, suppressed immunity, diarrhea, poor healing, stunted growth, hypogonadism, fetal growth failure, teratogenesis and abortion. Zinc deficiency is also known to be associated with various diseases such as malabsorption syndrome, chronic liver disease, chronic renal disease, sickle cell disease, diabetes, malignancy, neurodevelopment disorders and other chronic illnesses^1^,^2^,^3^,^4^,^18^,^19^,^20^,^21^,^22^. Zinc deficiency also causes significant impairment in adaptive and innate immune responses and promotes systemic immune dysfunction in older populations^2^,^4^,^7^,^23^,^24^,^25^,^26^.

Hair zinc concentration is commonly used in marginal zinc deficiency studies of children, and its usefulness has been documented in many industrialized countries including Canada and the USA^27^,^28^,^29^,^30^,^31^. Symptomatic zinc deficiency in infants was first reported in the early 1980s, with most cases occurring in breast-fed preterm infants^32^,^33^,^34^,^35^ because the zinc concentration of human milk is much lower than that of cow’s milk, and the demand for zinc increases rapidly in thriving preterm infants^36^.

In the present epidemiological study of 28,424 Japanese subjects, we demonstrated that the prevalence of zinc deficiency in infants is higher than that in adult and elderly subjects. In children aged 0–9 years, nearly one-third (male: 29.9%; female: 33.8%) of the subjects exhibited marginal to severe zinc deficiency. In infants aged 0–4 years, the incidence of zinc deficiency was particularly high: 36.5 and 47.3% in males and females, respectively (Fig. 5). Furthermore, a highly significant correlation (r = 0.298, p < 0.0001) between zinc concentration and age was observed in these children (Fig. 6). These results suggest that children, particularly infants, are susceptible to zinc deficiency.

The mechanisms that lead to zinc deficiency in infants may include unbalanced meals, lower absorption ability in the intestinal tract^37^,^38^, and low zinc concentration in maternal breast milk^39^. In addition, maternal dieting and cigarette smoking have been reported to be associated with lower zinc and higher cadmium and lead concentrations in neonates^40^. Toxic metals that have accumulated in the maternal bone matrix appear to be co-transferred with calcium and magnesium to fetuses and neonates through accelerated bone-resorption during pregnancy and lactation^40^,^41^,^42^.

Severe zinc deficiency in the rare inherited human disease acrodermatitis enteropathica has been reported to result from defective intestinal absorption of zinc due to mutations in the Zip4 transporter located in the intestinal tract^43^,^44^. Furthermore, recent genetic studies have indicated that mutations in the ZnT2 transporter gene in mothers produce zinc-deficient milk and cause breast-fed infants to develop a severe zinc deficiency^45^,^46^,^47^ that can be reversed by zinc replacement therapy^47^. These genetic factors, as well as various environmental factors, also contribute to some zinc deficiencies in infants.

In adults, highly significant (p < 0.001) inverse correlations between zinc concentration and age (r = −0.12 for males and −0.14 for females) were observed (Fig. 4A,B). In addition, a significant age-dependent increase in the prevalence of zinc deficiency was observed in males and females: from 2.0% to 15.1% by the 7^th^ decade of life and from 1.3% to 19.7% by the 8^th^ decade of life, respectively (Fig. 3). A study conducted in five European countries reported zinc deficiency in 31% of people over 60 years of age, with some country-specific differences in prevalence^48^. Another study of hospitalized elderly patients reported 28% zinc deficiency^49^. These findings indicate that elderly individuals are prone to zinc deficiency, even in developed countries.

It is interesting that male subjects over 85-years-old and females over 90 years old exhibited a low prevalence of zinc deficiency: 3.4% or less (Fig. 3). This finding that zinc deficiency is rare in the older age groups in both genders suggests that the mineral is essential for healthy aging and longevity and that zinc supplementation is probably effective and necessary for aging well.

Recently, dietary-restriction-induced zinc deficiency has been reported to up-regulate the intestinal zinc-importer (Zip4) and induce an increase in Zip4 protein present on the plasma membrane of enterocytes^50^. This adaptive response to zinc deficiency is known to increase the risk of high-uptake of toxic metals such as cadmium and lead. Thus, individuals with zinc deficiency are likely at an increased risk of absorbing large amounts of toxic metals and retaining them in their bodies. In fact, we have demonstrated a highly significant inverse relationship between hair zinc concentration and lead or cadmium concentration^20^. Thus, not only zinc deficiency itself but also the consequent increased risk of toxic metal burden seem to induce various physical and mental disorders^18^,^19^,^20^,^21^,^51^,^52^.

In conclusion, the present study of 28,424 Japanese subjects demonstrates that many cases of zinc deficiency are detected in infants and elderly individuals, indicating that these populations are prone to zinc deficiency. It remains to be established whether early intervention to correct zinc deficiency leads to normal development and health and to healthy aging and longevity.

## Methods

### Sampling and zinc analysis

After obtaining informed consent, scalp hair samples from 28,424 (male: 9,612; female: 18,812) subjects aged 0–100 years were collected from June 2005 to September 2007 (Table 1). Hair sampling was conducted by cutting hair as close as possible to the scalp of the occipital area.

A 75 mg hair sample was weighed into a 50 ml plastic tube and washed with acetone and then with a 0.01% Triton solution, in accordance with the procedures recommended by the Hair Analysis Standardization Board. The washed hair sample was mixed with 10 ml 6.25% tetra methyl ammonium hydroxide (TMAH, Tama Chemical, Kawasaki, Japan) and 50 μL 0.1% gold solution (SPEX Certi Prep, Metuchen, NJ, USA), and then dissolved at 75 C with shaking for 2 hours. After cooling the solution to room temperature, an internal standard solution was added. After adjusting its volume gravimetrically, the obtained solution was used for zinc analysis. The zinc concentrations were determined with inductively coupled plasma mass spectrometry (ICP-MS; 7500ce, Agilent Technologies, Santa Clara, CA, USA) by the internal standard method and expressed as ng/g hair (ppb) or μg/g (ppm)^18^,^19^.

The ethical committee of the La Belle Vie research laboratory reviewed and approved this study. The methods were carried out in “accordance” with the approved guidelines. Informed consent was obtained from all subjects. All of the data collected were held securely in such a form as to ensure anonymity.

### Statistical analysis

Because scalp hair mineral concentration follows a log-normal distribution, the log of the zinc concentration and the geometric rather than arithmetic mean were used to represent the hair zinc concentration. The relationship between age and zinc concentration was examined using Pearson’s correlation coefficient.

## Additional Information

**How to cite this article**: Yasuda, H. and Tsutsui, T. Infants and elderlies are susceptible to zinc deficiency. *Sci. Rep.* **6**, 21850; doi: 10.1038/srep21850 (2016).

## Figures and Tables

**Table 1:** Tested Japanese subjects (N = 28,424).

Age (years)	Female	Male
0–3	200	660
4–9	306	1,000
10–15	181	338
16–19	134	105
20–29	2,817	927
30–39	6,421	2,147
40–49	3,763	1,829
50–59	2,648	1,371
60–69	1,476	796
70–79	695	364
80–	171	75
Total	18,812	9,612
